# Nitric Oxide Photorelease from Silicone Films Doped with N-Nitroso BODIPY

**DOI:** 10.3390/jfb15040092

**Published:** 2024-04-02

**Authors:** Natalia A. Virts, Tatyana Yu. Karogodina, Mikhail A. Panfilov, Alexey Yu. Vorob’ev, Alexander E. Moskalensky

**Affiliations:** 1 Department of Physics, Novosibirsk State University, 630090 Novosibirsk, Russia; 2 Vorozhtsov Novosibirsk Institute of Organic Chemistry SB RAS, 630090 Novosibirsk, Russia

**Keywords:** nitric oxide, BODIPY, silicone, NO photodonor, photopharmacology

## Abstract

Nitric oxide (NO) is a unique biochemical mediator involved in the regulation of vital processes. Light-controllable NO releasers show promise in the development of smart therapies. Here, we present a novel biocompatible material based on polydimethylsiloxane (PDMS) doped with BODIPY derivatives containing an N-nitroso moiety that is capable of the photoinduced generation of NO. We study the green-light-induced NO-release properties with the following three methods: electrochemical gas-phase sensor, liquid-phase sensor, and the Griess assay. Prolonged release of NO from the polymer films after short irradiation by narrow-band LED light sources and a laser beam is demonstrated. Importantly, this was accompanied by no or little release of the parent compound (BODIPY-based photodonor). Silicone films with the capability of controllable and clean NO release can potentially be used as a highly portable NO delivery system for different therapeutic applications.

## 1. Introduction

Nitric oxide (NO) is a signaling gaseous molecule involved in a wide range of physiological and pathological processes [1,2]. Depending on its concentration, NO plays a role in pro- or anti-tumorigenesis, angiogenesis, metastasis, and immune responses. For example, NO, in the picomolar range, promotes tumor progression by conferring enhanced proliferative and angiogenic properties to cancer cells [3,4]. By contrast, a high concentration (≥1 μM) of NO can be helpful in cancer therapies by enhancing DNA, mitochondrial, and cell apoptosis and necrosis; suppressing tumor cell growth; reversing multi-drug resistance; and sensitizing tumor cells to chemotherapy, radiotherapy and immunotherapy [5,6,7]. NO has also been exploited as a vascular mediator for enhanced permeability in tumors, thereby improving the intratumoral delivery of nanomedicines [8,9]. Nitric oxide is also a potent pulmonary vasodilator for treating pulmonary hypertension and hypoxemia [10]. NO-donating molecules have broad-spectrum antimicrobial activity with similar potential as ROS, and even have a synergistic potential with ROS, to combat the growing trend of antibiotic resistance in bacterial infections [11,12,13,14,15].

In situ real-time NO generation could expand the potential and use of NO therapy. Various chemical species have the potential to be utilized as NO reservoirs for NO for controlled gas-phase NO delivery upon use of the appropriate stimulus/catalyst. For example, S-nitrosothiols can emit NO gas spontaneously by thermal decomposition in the presence of metal ion catalysts (e.g., Cu(I)) and/or reducing agents (e.g., thiols, ascorbate) [16,17], or by photolytic activation [18].

Photodynamically activated NO donor species, particularly those that may also generate ROS, are very attractive. Visible light absorption of these molecules is typically reached by conjugation of the NO-releasing moiety with chromophores such as rosamine and rhodamine [19,20,21,22,23,24,25], or carbon nanodots [26]. The BODIPY (4,4-difluoro-4-bora-3a,4a-diaza-S-indacene) chromophore could be superior as an antenna due to its unique photochemical properties, such as its narrow absorption bands with tunable wavelengths, which provide successful usage of these dyes in various scientific fields, from optoelectronics to life sciences (see [27] and references therein). The first BODIPY-based NO photodonor (NOBL-1) utilizing N-NO bond homolysis was reported by the Nakagawa group [28]. Extended work on BODIPY-based NO-releasing molecules was conducted by the Sortino group [29,30,31]. Several NO photodonors based on BODIPY [32] and aza-BODIPY [33] cores were developed by our group as well.

However, there are potential problems with the use of such NO photodonors in medicine. First, a high concentration of such compounds can only be reached in organic solvents, due to their aggregation in aqueous solutions. Second, all dyes inherently produce reactive oxygen species (ROS) under light, which exert toxic effects on cells. Third, the photodonor itself can be toxic or even cancerogenic since it contains N-Nitroso bond [34].

In our recent study, a compartment containing the solution of an NO photodonor was separated from the test compartment by a gas-permeable membrane, resulting in clean NO generation in the latter without any additional chemicals [33]. ROS also cannot pass the membrane due to their short lifetimes. NO mass transfer through different polymer films was demonstrated in [35]. Among different polymers, polydimethylsiloxane (PDMS) has demonstrated sufficient gas-penetration properties, which, together with its biocompatibility, gives this material the greatest potential for use. Moreover, PDMS was used as a matrix for the immobilization of NO (photo)donors. Frost et al. used incandescent light for the controlled release of NO from S-nitroso-N-acetylpenicillamine (SNAP)-derivatized fumed silica particles blended into PDMS [36]. Later, Gierke et al. [37] covalently linked SNAP to PDMS and used LED light with a 470 nm nominal wavelength for releasing the NO payload into an aqueous solution.

In this study, we focus on the investigation of BODIPY-based NO photodonors incorporated into the PDMS matrix. We show that the photodonors in this environment are capable of NO photorelease with better efficiency compared to organic solvents. We observed the prolonged release of photogenerated NO from the polymeric matrix due to passive diffusion, which also could be beneficial for NO-based therapy. Our results pave the way toward the development of a highly portable and inexpensive NO therapy system.

## 2. Materials and Methods

**Scheme 1:**
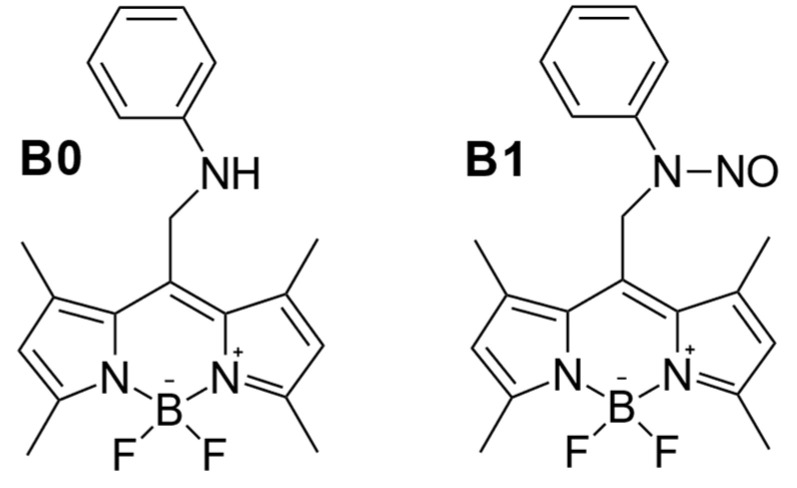
The structure of the photodonor.

**Synthesis of the photodonors.** In this work, we use a photodonor, the structure of which is shown in Scheme 1, designated by **B1**. We also used its analogue **B0**, which was synthesized independently for comparison with the photoproducts of **B1**. Synthesis of the compounds was done as described in [32].

**Figure 1:**
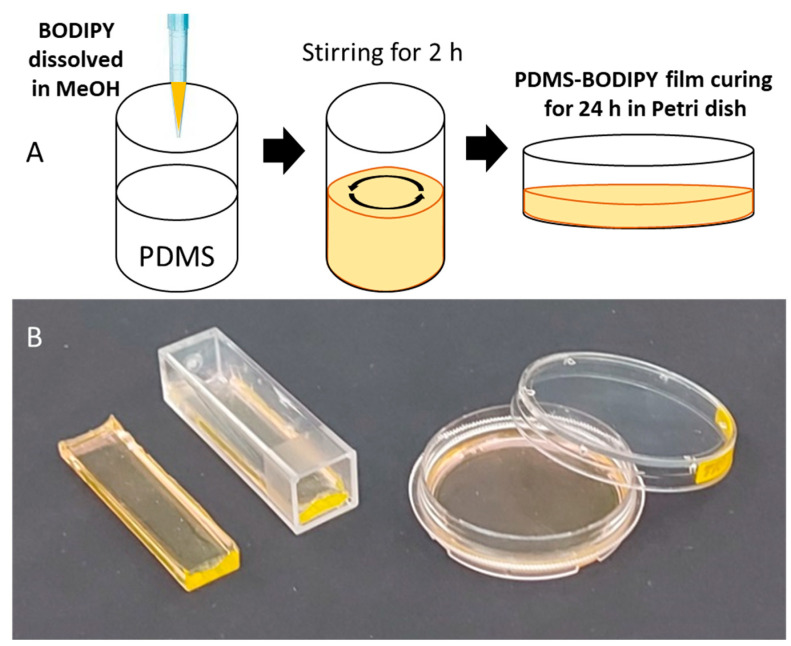
BODIPY-doped PDMS films fabrication process (**A**); and photo (**B**).

**Polymer film fabrication.** Dyes (NO photodonors) were dissolved in methanol and then mixed with the PDMS liquid monomer (base component of the Sylgard 184 kit) to a final concentration of 70 μM. Methanol makes the solution less viscous, and the dyes become equally distributed in the volume. It is important to mix the dye thoroughly with PDMS to avoid non-uniformity. In the next step, the curing agent was added to initiate polymerization and the mixture was left at room temperature for at least 24 h to allow for the evaporation of methanol and to complete polymerization. Flat strips of approximately 3 mm thickness were prepared in plastic cuvettes and round films were prepared in Petri dishes (Figure 1). The loading (calculated as the dye dry weight divided by weight of the polymer) was about 0.002%. The prepared films were stored at room temperature in the dark. During the whole fabrication process, the light exposure of the doped films was minimized by working in dimmed light conditions.

**Spectroscopy study**. UV–VIS transmittance of the PDMS films was measured with a spectrophotometer Shimadzu UV-1900 (Kyoto, Japan). The photolysis was conducted with high-power LEDs (500 nm central wavelength) or with a 488 nm, 50 mW continuous laser (CrystaLaser DL488-050, Reno, NV, USA). Configuration of the light sources was adapted to the shape of the PDMS films used in the different experiments. In experiments involving the laser, the mean laser power was modulated by pulse-width modulation using an Arduino microcontroller connected to the modulation input of the laser controller. The diameter of the laser beam is 2 mm, which coincides with the diameter of the electrochemical sensor used in these experiments, which is why we did not use focusing.

**Griess test.** A colorimetric Griess assay was used in this study to determine the concentration of nitrites in the sample [38,39]. The Griess assay (10% by weight) was prepared by diluting commercially available Griess reactive powder (Component-Reaktiv, Moscow, Russia 3579.1000) with 12% percent acetic acid followed by filtration from the insoluble precipitate. The calibration procedure was performed using freshly prepared NaNO_2_ solutions as a standard. According to the procedure, 1.5 mL of the analyzed solution was mixed with 1.5 mL of the Griess assay and incubated for 40 min. Then, the absorption spectrum was measured. During the reaction of the Griess assay with nitrites, optical density increases at 500–550 nm, with maximum at 526 nm. The Griess assay was used to estimate the release of nitric oxide from the PDMS films into the solution. For this, PDMS films were immersed in a water solution containing the Griess assay (1:1), then illuminated by green light and incubated for 40 min. Then, the test solution removed and placed in a quartz cuvette for measuring of the absorption spectrum and for quantification.

**NO gas sensor.** We used the amperometric NO gas sensor Alphasense NO-B4 equipped with a custom-made potentiostatic circuit, designed as described in [40]. The operational amplifiers for bias voltage control and current-to-voltage conversion were chosen, as described in [41] (Section S1: Hardware considerations). The resulting output was digitized using a 16-bit ADC ADS1115 and transmitted to a PC by an Arduino Nano microcontroller for plotting and data logging.

**Electrochemical detector.** The WPI ISO-NOP nitric oxide sensor was used. We used TBR-1025 for the current-to-voltage conversion and amplification. Then, the output voltage was digitized using a 16-bit ADC ADS1115 and transmitted to a PC by the Arduino Nano microcontroller for plotting and data logging. The same microcontroller was used to manage the intensity of the laser beam, which traversed the sample directly under the sensor tip. The PDMS film was immersed in water and placed so that it was in contact with the sensor tip. The sensor and the sample were inside a grounded Faraday cage to avoid the influence of external electric fields.

## 3. Results and Discussion

**Figure 2:**
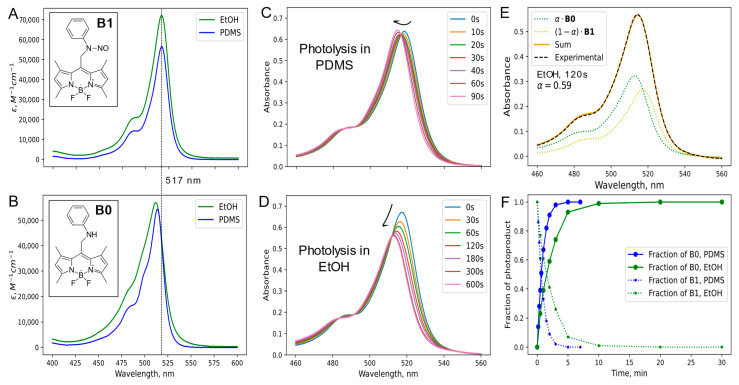
(**A**,**B**) absorption spectra in EtOH and PDMS of **B1** and **B0**, respectively; (**C**,**D**) changes in the absorption spectra of **B1** during photolysis; (**E**) example of the decomposition of absorption spectra to the spectra of the initial and final products (photolysis in EtOH for 2 min); and (**F**) kinetics of the transition from initial to final product in EtOH and PDMS.

PDMS is a unique material in many respects, including for its biocompatibility and optical properties. It usually acts as a porous elastic solid, making it a convenient material for the incorporation of small molecules and dyes. However, the spectroscopic properties of dyes in PDMS may change significantly, which may impede the desired action; therefore, we first tested the properties of NO photodonors in such an environment. Figure 2A shows the absorption spectra of **B1** in EhOH and the PDMS film prepared as described in Methods. The spectra are similar in shape and have maxima at the same wavelength—517 nm, although the maximal extinction coefficient ε is higher in ethanol (Table 1). In contrast, the maximal extinction coefficient of **B0** is almost the same in EtOH and PDMS, while the maxima are situated at slightly different wavelengths. Interestingly, the absorption spectrum of **B0** is narrower in PDMS.

The results in Figure 2B were obtained with independently synthesized compound **B0**, which corresponds to the main photoproduct of **B1** only hypothetically. Therefore, we performed the photolysis experiment, measuring the absorption spectra after each act of high-intensity green-light illumination. Figure 2C shows the resulting changes as follows, which are in agreement with the hypothesis: the maximal wavelength shifted from 517 to 514 nm, while the height of the spectra is almost unchanged. For the comparison, we reproduced the experiment in EtOH [32], where the spectral shift was accompanied by a decrease in optical density (Figure 2D). This is in agreement with the fact that ε for **B1** is significantly higher than that for **B0** in EtOH, while in PDMS, the extinction coefficients are almost the same.

To analyze the dynamics of photoinduced transformation, we used the mathematical approach described below based on two assumptions. The first is that the sample contains only two species, namely, the initial compound and the photoproduct. The second is that their absorption spectra are additive, i.e., the experimental spectrum S is expressed as

(1) $$S=\alpha S_{final}+\left(1-\alpha \right)S_{initial},$$

where α is the fraction of the photoproduct depending on time, and S, $S_{initial}$ and $S_{final}$ are vectors containing experimental data at intermediate, initial, and final time points, respectively. Then, the fraction α can be found by linear least-square optimization as follows:

(2) $$\left\|S-\alpha S_{final}-\left(1-\alpha \right)S_{initial}\right\|\to min,$$

(3) $$\frac{\partial }{\partial \alpha }\left[{\left(S-S_{initial}\right)}^{2}-2\alpha \left(S-S_{initial}\right)\cdot \left(S_{final}-S_{initial}\right)+\alpha^{2}{\left(S_{final}-S_{initial}\right)}^{2}\right]=0,$$

(4) $$\alpha =\frac{\left(S-S_{initial}\right)\cdot \left(S_{final}-S_{initial}\right)}{{\left(S_{final}-S_{initial}\right)}^{2}},$$

where ⋅ denotes the dot product of two vectors. Figure 2E shows an example of the decomposition of the absorption spectrum at an intermediate time point (120 s in EtOH) according to Equations (4) and (1). Good agreement of the experimental spectrum and the sum of the two basis vectors shows that the assumptions are reasonable.

Figure 2F shows the dependence of fraction α on time in EtOH and PDMS. It can be seen that the transformation is twice as fast in PDMS. As the absorbance and other parameters in both experiments were the same, we conclude that it is the microenvironment that influences the photodecomposition speed. It is usually believed that photoinduced electron transfer is the main mechanism of NO photorelease from BODIPY-based compounds. The excited dye core absorbs the electron from N-NO, followed by the homolysis of the (N-NO)^+^ bond with NO release and the formation of an aminyl radical. Our results indicate that the process occurs more efficiently in a rigid PDMS matrix. This fact requires additional discussion.

Rigid matrices can inhibit processes which require diffusion, such as photoinduced electron transfer between donor and acceptor molecules. This is due to the fact that the mobility and flexibility of dyes incorporated into a polymer are restricted. However, in the case of tightly tethered donor and acceptor groups (intramolecular electron transfer) diffusion is not required. Moreover, the polymeric environment in some cases could actually promote this process by several means. First, it can influence the electronic structure of the molecule and the mutual orientation of donor and acceptor groups within a molecule, optimizing the electronic coupling between them. Second, it can provide a protective environment for the stabilization of reactive intermediates, such as radical ions, that are formed during NO photorelease. Third, the unproductive energy decay channel into molecular motility is blocked, resulting in the increased efficiency of photoinduced processes. In fact, this effect was independently demonstrated in conformationally restrained boron-methylated BODIPY photocages, where the introduction of rigid structures into the molecule led to the increase in photorelease quantum efficiency [42]. The several-fold increase of efficiency was demonstrated for NO photodonors incorporated into methoxy-poly(ethylene glycol)-polycaprolactone [43] and Pluronic block-copolymers [44].

For the case of the described polymer films, NO is generated inside a bulk material. Although PDMS is known to be well-permeable for small gaseous NO molecules [35], we performed several experiments to prove the NO release and evaluate its dynamics.

**Figure 3:**
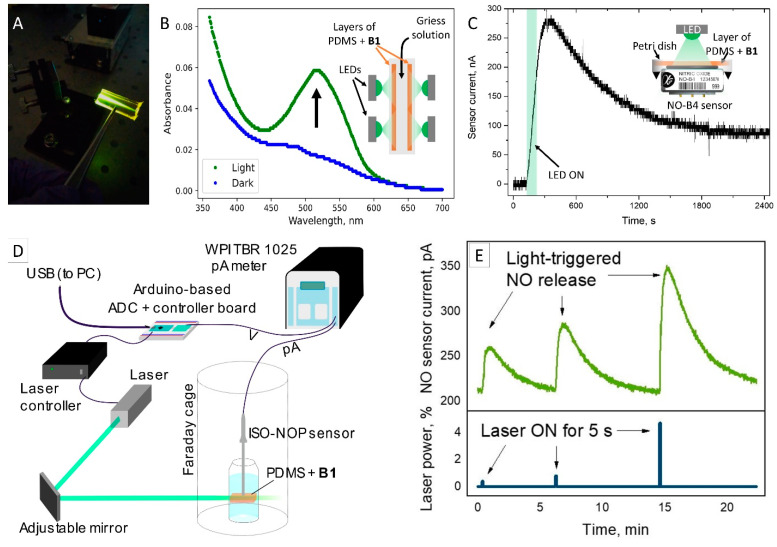
(**A**) Photo of the laser beam passing through **B1**-doped PDMS film; (**B**) Griess assay showing the increase in nitrite concentration in a sample after irradiation of PDMS films by green light; (**C**) dynamics of gas-phase NO release from PDMS film monitored with selective NO-B4 sensor; (**D**) scheme of experimental setup for measuring laser-induced NO release into aqueous solution; and (**E**) prolonged light-induced, power-dependent release of NO into the aqueous solution monitored by an electrochemical sensor.

NO is rapidly oxidized to nitrites and nitrates. Total NO production can be evaluated by tracing the concentration of these products in a sample. The Griess assay is commonly used for this purpose. We used a freshly prepared Griess assay, as described in Methods, to evaluate nitrite yield in the sample. The test solution containing the Griess solution was prepared in a standard optical cuvette. PDMS films were immersed in a test solution and illuminated by green LEDs, as shown in the Figure 3B. The illumination time was 6 min to achieve total photolysis. Then, the test solution was moved to another cuvette and the absorption spectra of the sample was measured. Compared to the initial spectrum, the increase in absorption at ~526 nm is clearly visible. According to the calibration, this increase corresponds to 0.72 µM of nitrites. We also performed the control experiment in the same conditions but without the Griess assay and observed growth of absorbance at 517 nm by ~0.01, probably corresponding to slow dye leakage from the PDMS film. However, the amount of dye was small and cannot fully account for the growth of absorbance of the Griess assay. Figure 3B.

The gas-phase NO release from PDMS films was demonstrated in [10] using the selective Alphasense NO-B4 sensor. We also conducted experiments with this sensor to test the effect in our conditions. Figure 3C shows the design of the experiment. The 3 cm-diameter round film doped with **B1** was placed directly upon the sensor. The release of NO gas is indicated by the increase in the current, which started synchronously upon illumination and lasted after the LED was switched off, indicating the diffusion of NO through the PDMS matrix. Given the manufacturer-provided sensitivity of the sensor 500–850 nA/ppm, the maximal detected current of ~300 nA corresponds to 0.3–0.6 ppm NO.

Next, we tested the release of NO into an aqueous solution using the modified setup that we described earlier [33]. Briefly, a 488 nm laser was used to illuminate the film, and the NO release was monitored with an ISO-NOP amperometric sensor (Figure 3D). The setup was connected to the PC for control and data logging.

The PDMS film was immersed in water and placed so that it was in contact with the sensor tip. The laser beam was set up so that it traversed the sample directly under the sensor tip and their diameters are the same (2 mm). The laser is controlled by user from the PC, and can be turned on/off with the desired mean power (modulated using PWM). As shown in Figure 3E, short, 5 s laser pulses triggered slow NO release, which lasted for minutes. The intensity of the sensor response depended on the laser power, which was from 1 to 5% of maximum, corresponding to 0.5–2.5 mW. Interestingly, there was no response if the sensor tip was not in direct contact with the **B1**-doped PDMS film. We also performed control experiments with pure PDMS film and film doped with **B0** and observed no response.

**Figure 4:**
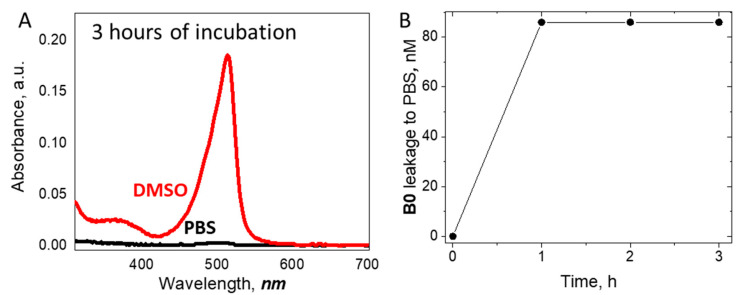
(**A**) Absorption spectra of cuvettes with DMSO (red line) and PBS (black line) after incubation of PDMS film doped with the dye **B0**; and (**B**) time dependance of the dye leaking into PBS.

Finally, as the encapsulation of the NO photodonor in the polymer was non-covalent, we evaluated the leaching process, which is a common drawback of such systems. Stability of the doped material was tested in DMSO and phosphate-buffered saline (PBS, pH 7.4) as a model of the physiological conditions. The polymer film containing **B0** was placed into the cuvette with DMSO or PBS for incubation, and the liquid was periodically tested for dye content. Leaching was indeed observed in DMSO; however, the dye leakage to PBS was insignificant and completely stopped after 1 h of incubation (Figure 4A,B).

This result is in line with the known hydrophobicity of PDMS, which prevents wetting of the material with aqueous solvents and hinders the leaching process. The observed dye leakage was probably due to a minor number of molecules located at the film surface, which were the only ones able to dissolve in water. In contrast, DMSO is capable of penetration to the PDMS’s internal pores, which explains the extraction of dye.

To sum up, this paper presents the following results.

First, following the described preparation procedure, we obtained the PDMS films with uniformly distributed dyes. The absorption spectra of compounds used in EtOH and PDMS were similar in shape and had maxima at the same wavelengths, although the extinction coefficient of the photodonor was higher in ethanol (Table 1). Interestingly, the absorption spectrum of the compound B0 was narrower in PDMS, which may indicate a lower fraction of dye aggregates. These results indicate that BODIPY dyes are well-soluble in silicone/PDMS and that the latter can be used as a solid-state matrix for the incorporation of these dyes in optoelectronic devices and biomedical applications.

Second, we studied the photolysis of the NO donor **B1** in the PDMS matrix. The dynamics of the absorption spectra were the same as those in the organic solvent; the maximal wavelength shifted from 517 to 514 nm. Interestingly, the decrease in optical density, which accompanied the experiment in EtOH, was not observed in PDMS. This is agreement with the fact that the extinction coefficient for **B1** is significantly higher than that for **B0** in EtOH, while in PDMS, the extinction coefficients are almost the same. We showed that the dynamics of spectral change are several times faster in PDMS than in organic solvents. This indicates that the solid-state polymeric environment influences the photochemistry of the NO photodonor, enhancing the probability of photodecomposition.

Third, our experiments indicate that NO released inside the polymer is able to diffuse to the outside. To show this, we used several approaches to detect the release of NO from the PDMS film. A Griess reagent was used to estimate the downstream products of nitric oxide in a solution surrounding the film. The electrochemical sensor ISO-NOP was used to directly measure the NO release dynamics using an amperometric technique. Finally, a gas-phase NO sensor was used to confirm its release from the PDMS film to the surrounding air.

Taken together, the described results confirm the photoinduced release of NO from a polymeric PDMS matrix containing the BODIPY-based donor. Apart from the unique capability of precise control of the effect by light, the solid-state film provides several advantages as a container of the NO photodonor as compared to the solution in an organic solvent. In particular, the photodecomposition of the donor occurs more rapidly inside the film as compared to the organic solvent. Solid-state film is more convenient for incorporation in various devices and theranostic systems. It prevents the contact of the dye itself with the surrounding media (e.g., biological tissues). It completely eliminates issues related to the potential toxic and cancerogenic properties of the photodonor. Moreover, reactive oxygen species, which are inevitably formed during photoexcitation, are not able to exert toxic effects because they cannot escape the matrix during their short-lived excited state. At the same time, the relatively long-lived NO molecule is able to penetrate through the polymeric matrix and act outside the film. We observed the prolonged release of photogenerated NO from the polymeric matrix due to passive diffusion, which is beneficial for NO-based therapy. Our results pave the way toward the development of a highly portable and inexpensive NO therapy system.

Further studies will be aimed at designing such systems, which should incorporate the NO donating polymer film, a light source, and a control unit. Among the most intriguing applications for these systems are implantable devices such as vascular grafts. Indeed, some attempts to use NO-donating graft materials were made; it was shown that such materials help to inhibit platelet adhesion and restenosis [45]. In this respect, the development of near-infrared NO photodonors is of great interest, because this type of radiation penetrates deep into tissues. Alternative techniques can rely on the up-conversion materials, which are able to use longer-wavelength radiation (such as near-infrared) for the emission of visible light or the direct transfer of energy to the BODIPY energy levels. Another potential research direction is related to the enhancement of NO generation in terms of speed or required concentrations. For example, recent studies [46,47] showed the possibility of enhancing NO generation in the presence of localized plasmon resonance, which non-radiatively activates the NO donor. This effect has the potential to be realized in other systems with NO donors, since plasmon resonance has an effect on the optical properties of organic compounds [48].

## 4. Conclusions

In this paper, we report on novel material based on a PDMS film doped with BODIPY-based NO photodonor. Using several methods, we show that the unique environment inside the films increases the efficiency of the photodonor and provide prolonged NO release triggered by short light pulses. Given the well-known biocompatibility of PDMS, the reported material is suitable for various bio-applications and for the future design of smart therapies.

## Figures and Tables

**Table 1:** Spectroscopic properties of **B0** and **B1**.

		*λ*_max_, nm	ε, M^−1^ cm^−1^
**EtOH**	**B0**	512	57,100
	**B1**	517	72,100
**PDMS**	**B0**	514	54,400
	**B1**	517	56,500

## Data Availability

Data will be made available on request.
